# Rapid start antiretroviral therapies for improved engagement in HIV care: implementation science evaluation protocol

**DOI:** 10.1186/s12913-023-09500-w

**Published:** 2023-05-17

**Authors:** Beth Bourdeau, Starley B. Shade, Kimberly A. Koester, Greg M. Rebchook, Wayne T. Steward, Bruce M. Agins, Janet J. Myers, Son H. Phan, Marlene Matosky

**Affiliations:** 1 Division of Prevention Science, University of California, San Francisco, San Francisco, CA USA; 2 Division of Policy and Data, Health Resources and Services Administration HIV/AIDS Bureau, Rockville, MD USA

**Keywords:** HIV, Rapid start antiretroviral therapy, Implementation science, Evaluation, Mixed methods, Learning collaborative, United States

## Abstract

**Background:**

In 2020, the Health Resources and Services Administration’s HIV/AIDS Bureau funded an initiative to promote implementation of rapid antiretroviral therapy initiation in 14 HIV treatment settings across the U.S. The goal of this initiative is to accelerate uptake of this evidence-based strategy and provide an implementation blueprint for other HIV care settings to reduce the time from HIV diagnosis to entry into care, for re-engagement in care for those out of care, initiation of treatment, and viral suppression. As part of the effort, an evaluation and technical assistance provider (ETAP) was funded to study implementation of the model in the 14 implementation sites.

**Method:**

The ETAP has used implementation science methods framed by the Dynamic Capabilities Model integrated with the Conceptual Model of Implementation Research to develop a Hybrid Type II, multi-site mixed-methods evaluation, described in this paper. The results of the evaluation will describe strategies associated with uptake, implementation outcomes, and HIV-related health outcomes for patients.

**Discussion:**

This approach will allow us to understand in detail the processes that sites to implement and integrate rapid initiation of antiretroviral therapy as standard of care as a means of achieving equity in HIV care.

Of more than 1.2 million people in the United States with human immunodeficiency virus (HIV), approximately 87% are aware of their HIV diagnosis [1], but only 76.0% of those are engaged and 57.8% retained in medical care [2]. Often, there is a delay between HIV diagnosis and initiation of antiretroviral therapy (ART), due to unnecessary complexities in the care delivery system, increasing the likelihood that patients fall out of care. However, initiation of ART within a week of HIV diagnosis for newly diagnosed individuals or those re-entering care reduces the time to viral suppression [3].

In 2020, the Health Resources and Services Administration’s (HRSA) HIV/AIDS Bureau Ryan White HIV/AIDS Program (RWHAP) Part F – Special Projects of National Significance (SPNS) funded the “Building Capacity to Implement Rapid ART Start for Improved Care Engagement in Ryan White HIV/AIDS Program” Initiative, with its Evaluation and Technical Assistance Provider (ETAP) awarded to the University of California, San Francisco (UCSF) Center for AIDS Prevention (CAPS). The ETAP’s role is to provide support to, and evaluate the implementation of, rapid ART start in 14 RWHAP settings across the U.S. with the goal of developing an implementation blueprint for integrating immediate access to ART in HIV care settings. In this manuscript, we describe the approach to evaluate whether and how strategies undertaken improve: organizational capacity, delivery of rapid ART start, and patient outcomes associated with engagement in care.

## Background

In 2013, the Zuckerberg San Francisco General Hospital’s Ward 86 HIV clinic implemented services for providing ART on the same day as HIV diagnosis [4]. Between 2013 and 2017, 96% of patients accepted immediate ART and 96% achieved viral suppression within 1 year of starting ART [5, 6]. Results in comparable programs [7, 8] prompted the World Health Organization (WHO) to recommend universal “test and treat” [9] and was soon followed by other HIV clinical care professional organizations [10]. Current U.S. Department of Health and Human Services guidelines now endorse treatment as early after diagnosis as possible, same day if feasible [11], as do the European AIDS Clinical Society (EACS) and International Antiviral Society (IAS) [12].

ART initiation is considered “rapid” when treatment begins as soon as possible after a positive HIV test [13] though at present, there is no unified, evidence-based definition for rapid ART start nor standard protocols for implementing or evaluating rapid ART start services [12]. Regardless, evidence suggests that shortening the time between diagnosis and ART initiation is linked to increased retention in HIV care and decreased time to viral suppression [14–19], higher quality of life [20], lower risk of partner infection [21, 22], better health outcomes and slower disease progression [23–25], and lower mortality. [26]

Delivery of rapid start ART services requires organizational *reorganization* of procedures, multidisciplinary coordination, and consolidation of patient services, including clinical evaluation, education, counseling, ascertainment of healthcare coverage and laboratory testing, all fit into a 2- to 3-hour initial clinic visit [5, 27]. *Time* is required to arrange healthcare coverage if needed, initiate work with patients for psychological and social stabilization, provide education and counseling about HIV and ART, and select an appropriate ART regimen for the patient [4, 28]. Specific *data* collection strategies have also emerged as important tools for driving implementation, assessing success, and facilitating sustainability [6].

Barriers to rapid ART start service deployment exist on multiple levels [29]. Identified barriers for people with HIV include poverty and its sequelae [30–34], behavioral health conditions [35], HIV treatment literacy [36], stigma [36, 37], and disease co-infection [38, 39]. At the organizational level, barriers include HIV workforce challenges [40–42], data sharing restrictions [43, 44], and healthcare coverage issues [28, 45]. Institutional inertia, access to same-day insurance, personnel [46], clinic capacity issues, inefficient referral networks, and lack of culturally-appropriate care [47] are likely to affect access to rapid ART start services. Strains on the HIV care workforce raise questions about the long-term sustainability; provider shortages and increased demand on linkage to care or navigation services are also challenges [48]. Costs for initiation of rapid ART start services and processes, patient load, and the variety of third-party payers can all affect rapid ART start implementation [49]. In New Orleans, the high percentage of the patient population already enrolled in Medicaid at the time of linkage was an important determinant of success [8]. Implementation within states that did not expand Medicaid can face significant challenges.

### Rapid start initiative description and evaluation

The RWHAP SPNS Rapid Start ART initiative is a three-year project and funds 14 RWHAP implementation sites initiating and/or expanding rapid ART initiation. Sites differ in the context in which those services are provided (university hospitals, federally qualified health centers, community-based organizations), and where they are located throughout the U.S. (rural and urban settings). To support implementation, we have utilized the Institute for Healthcare Improvement’s Collaborative Model [50] (colloquially known as “learning collaboratives”) as a primary technical assistance mechanism [51]. We have used a similar approach on multiple prior HIV service projects [52, 53]. Each of the implementation sites work with an assigned coach with expertise in quality improvement (QI) methods in HIV care delivery; participate in initiative-wide, two-day Learning Sessions three times a year; and attend webinars and cohort meetings that include peer learning and/or experts in rapid ART start. We have developed and are executing an implementation study as our multi-site evaluation, based on an integration of implementation research frameworks.

## Methods/design

### Evaluation framework

The evaluation is informed by both the Dynamic Capabilities Framework [DCF [54, 55]] and the Conceptual Model of Implementation Research (the Proctor Model) [56, 57]. DCF recognizes that factors affecting healthcare systems are in flux and implementation of any one change is going to be influenced by factors at multiple levels. We have applied this framework previously to our evaluation of health information systems in HIV clinical settings and studies of practice transformations [58]. The Proctor Model focuses on the importance of implementation strategies as facilitators of program implementation and success, and on the assessment of implementation, service, and client-level outcomes.

**Fig. 1:**
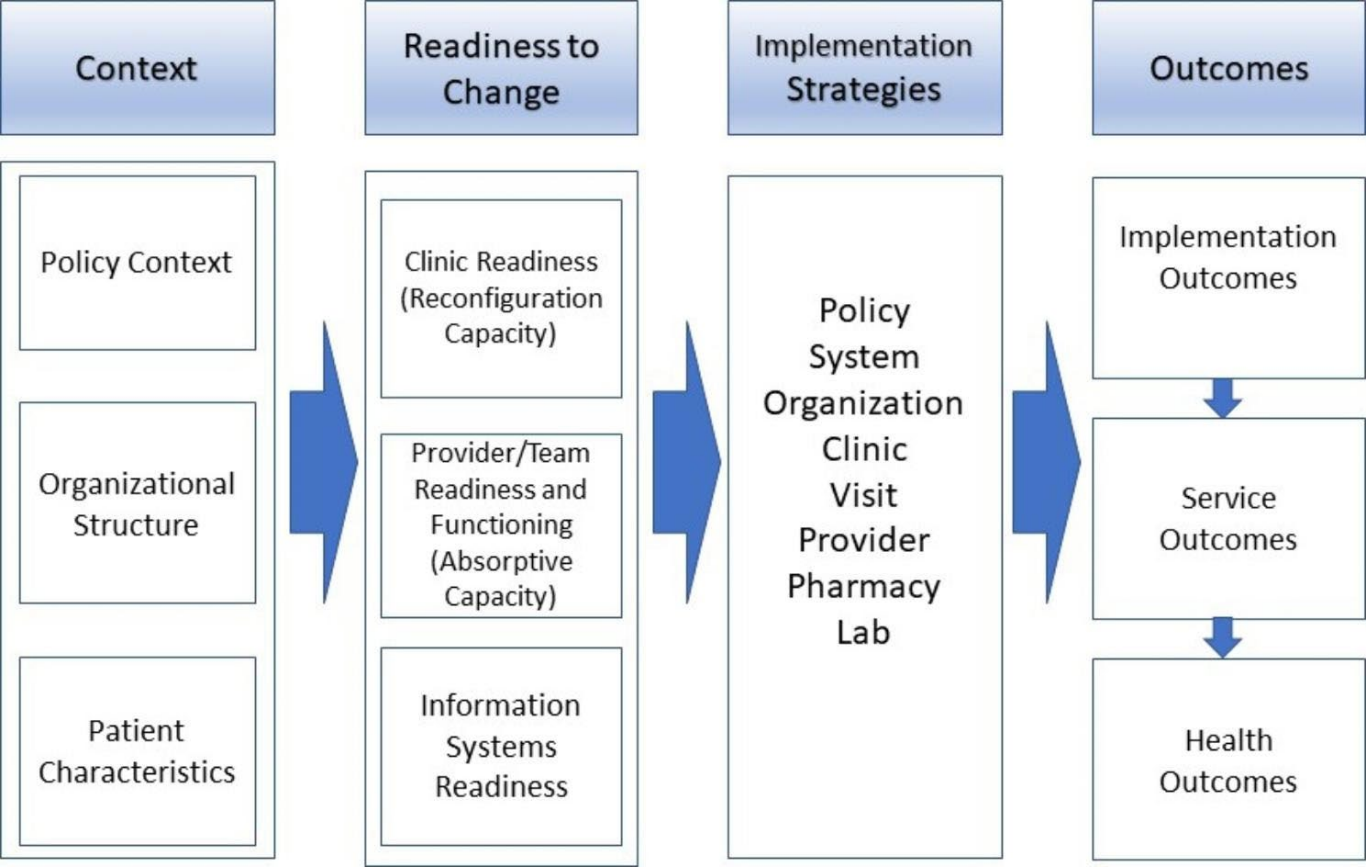
Rapid ART Start Initiative Implementation Conceptual Framework (Dynamic Capabilities and Proctor Model)

Our combined approach from DCF (Context and Readiness to Change) and the Proctor Model (Implementation Strategies and Outcomes) is shown in Fig. 1. *Clinic level readiness* is known as “reconfiguration capacity”: the ability of an organization to modify workflows, accommodate practice change, and readiness to reconfigure the process within the overall organizational system. By contrast, *provider level readiness* is the “absorptive capacity” of care providers: willingness to participate in change, beliefs about that change, and ability to take up the change. Finally, because of the importance of data in change processes, *information systems readiness* refers to the information collected in a health information system that must be configured to collect the data needed for successful implementation. Clinic, provider, and information system readiness influence the choice and use of strategies that can then support implementation of rapid ART start services and achieve desired implementation outcomes. *Implementation Strategies* are methods used to enhance the adoption, implementation, and sustainability of a service approach; *Implementation Outcomes* are effects of deliberate and purposive actions to implement a new intervention; *Service Outcomes* are ideal qualities of health care; and, *Health Outcomes* are impacts of implementation on the patients [57].

### Procedures and measures

We designed a mixed-methods approach to the evaluation (see Table 1), using an interrupted time series approach for the quantitative components and on-going qualitative data collection, designed to minimize data collection burden on site staff and requiring no collection directly from patients. All methods and procedures were subject to peer review prior to the funding decision by HRSA HAB and received Exempt Certification by the Institutional Review Board of the University of California, San Francisco in March 2021 (#20-32492).

**Table 1:** Evaluation Research Constructs

Model Domain	Constructs Assessed Within Domain	Data Source
Policy Context	Medicaid Expansion Ability to use starter packs Insurance accepted Availability of ADAP (or similar)	Organizational Assessment
Organizational Structure	Placement within larger institution (e.g., clinic part of a large hospital) Organizational size Degree of independence/authority within the organization	Organizational Assessment
Patient Characteristics	Demographic characteristics Behavioral health measures	Medical Record Data
Reconfiguration Capacity (Clinic Readiness)	Leadership vision and engagement Clinic’s current staffing structure, including total number of providers and panel sizes of primary care providers Clinic’s current practice workflows Resources (money, time, staff) for implementing changes (e.g., starter packs, resources for wrap-around services)	Organizational Assessment Cost Assessment Implementer Assessment Document Review
Absorptive Capacity (Provider Team Readiness)	Team functioning Knowledge of proposed changes Capacity to overcome barriers (insurance) Willingness to change Self-efficacy about implementing changes Beliefs about efficacy of changes Need for training Burnout	Implementer Assessment Document Review
Information System Readiness	Types of information currently stored in the EHR (collection of contact with patients) Access and privileges of various settings/providers (e.g., ability to share data between testing, care, and pharmacy; read EHR data; ability to input data) Capacity (personnel, skills) to modify system as needed	Organizational Assessment Document Review
Implementation Strategies	Rapid Start Service Characteristics Implementation of rapid ART start protocols	Medical Record Data Organizational Assessment Implementer Assessment
Implementation Outcomes	Acceptability (provider) Adoption (provider and clinic system) Appropriateness (provider and clinic system) Feasibility (provider) Fidelity (provider and clinic) Cost (clinic)	Medical Record Data Organizational Assessment Implementer Assessment Cost assessments
Service Outcomes	Effectiveness and Equity Demographic/patient characteristics by: • ART among persons in HIV care • Linkage to HIV care • Engagement in HIV care • Retention in HIV care Timeliness • Time to linkage to HIV care • Time to ART initiation	Medical Record Data
Health Outcomes	Viral load suppression • Time to viral load suppression • Durable viral load suppression	Medical Record Data

### Data Collection

#### Qualitative Document Review

Document review is a continuous process to assess rapid ART start implementation. Secondary documents include: grant applications; reports from annual site visits; monthly site monitoring call notes; materials created in the coaching and Learning Collaborative process; technical assistance requests; process maps; and documentation of QI projects. We use a template to guide extraction of relevant information from each source document. Every six to eight months, the ETAP conducts a content analysis of data abstracted to produce a longitudinal analysis of implementation progress for each site.

##### Domains

Document review allows us to identify *clinic readiness* via information contained within implementation plans, such as grant proposals, coach meeting notes, or notes from monthly monitoring calls that take place with members of our team and HRSA Project Officers. *Provider team readiness and functioning* is captured in coaches’ notes as they attend to dynamics among the implementation team. *Information systems readiness* is noted by coaches in the processes by which implementation sites modify their electronic health record (EHR)/electronic medical record (EMR) to generate data needed for QI. Barriers and facilitators to implementation of selected strategies are extracted on a routine basis.

#### Qualitative Implementer Assessment

We conduct open-ended assessments with up to 10 key stakeholders and implementers per site. Participants include those directly involved in implementation (i.e., test counselors, case managers, navigators, social workers, benefits counselors, registered nurses, prescribers) and any individuals in key leadership positions. These qualitative surveys are self-administered via Qualtrics whereby open-ended responses can be entered. These assessments take place twice: at implementation mid-point reflecting over the first year (summer 2022) and toward the end of the initiative (summer 2023).

##### Domains

The assessments are designed to capture information related to *provider readiness* and *implementation outcomes* (acceptability, appropriateness, adoption, feasibility). In addition, these assessments capture information on *implementation strategies*. These questions ask for the staff members’ perceptions at the time of the administration as well as retrospectively.

#### Quantitative Organizational Assessments (OA)

The Organizational Assessment is orally administered by ETAP staff at baseline and prior to each Learning Session. Organizational assessments are emailed to the Project Director ahead of time to allow preparation of the information; the Project Director has the discretion to determine attendance at the administration. Answers are tracked across assessment waves to capture change over time.

##### Domains

The OA serves to capture information on *context* (policy, organizational structure, and patient characteristics), including resources needed and used (infrastructure, equipment, partnerships, staffing), and activities (services provided, training, and team management) [59]. It also addresses clinic readiness (*reconfiguration capacity*), using questions about updated workflows and policy decisions, as well as the structure (availability of capital, time and resources for innovation) and Culture (idea solicitation, collaboration, risk tolerance) subscales of the Accelerated Healthcare Innovation Capacity Scale [60]. Questions in the OA include Baseline *Information Systems Readiness* assessments of the Electronic Health Record in order to understand capacity and access issues and the degree to which the system can be modified, if necessary, to track implementation and conduct QI projects. The concepts of acceptability, adoption, and feasibility are assessed using scales developed specifically for longitudinal documentation: the Acceptability of Intervention Measure (rapid ART start is welcomed and appealing), Intervention Appropriateness Measure (rapid ART start is suitable and applicable), and Feasibility of Intervention Measure (rapid ART start is easy to use and implementable) [61].

#### Quantitative Medical Record Data

Medical data are collected on *all* rapid start ART-eligible patients at the implementation sites, regardless of when/if they actually start ART. “Rapid-eligible patients” are as follows: (1) *newly diagnosed* includes any person with a new positive HIV rapid, confirmatory, or detectable viral load test result within 12 months of the site becoming aware of their positive status; (2) *new to care* includes any person diagnosed with HIV greater than 12 months prior who has not previously attended a HIV care medical visit or has never been on ART; and (3) *out of care* includes any person diagnosed with HIV with previous engagement in primary HIV care but who has not had a medical visit, ART prescription, or laboratory test result for greater than 12 months, and has agreed to return to care.

The patient clinical data serve two purposes in this initiative: (1) generation of quality measures for review and reflection within the Learning Collaborative including QI projects; and (2) transformation into coded patient-level records for inclusion in the multisite evaluation. The Excel workbook into which the data are entered autogenerates reports/graphs used in coaching sessions and presentations at Learning Sessions. The same workbook autogenerates de-identified, coded data for inclusion in the multisite evaluation. Sites collect and submit data from patient medical records on a monthly basis order to capture information on all eligible patients and computed quality measures variables.

Service launch commenced with the first Learning Session (March 30 and April 7, 2021), establishing the pre-implementation **retrospective** baseline which includes all rapid-eligible patients from April 1, 2020, through March 31, 2021. This assesses each site on their performance prior to their substantive work within the Learning Collaborative. Sites continue to collect the same data points on all rapid-eligible patients for the **prospective** data collection, which includes all rapid-eligible patients seen for their initial visit between April 1, 2020, and March 31, 2023, with follow-up data collected through June 30, 2023.

##### Domains

From this information, an overall assessment of *service outcomes* can be determined, including provision of rapid start ART to all who could benefit (*effectiveness*) and demonstration that delivery does not vary by patient characteristic (*equity*). The amount of time elapsed between points of contact reveals any delays in treatment (*timeliness*). Finally, we can determine if rapid start service provision results in better *health outcomes*, namely viral suppression, and durable viral suppression.

#### Quantitative Costing Data

Costing workbooks are designed by the ETAP and utilized in several previously funded RWHAP SPNS initiatives. As with the Organizational Assessment process, the Project Director is sent an advance copy of the costing template to assemble the information requested. The workbook is then submitted using an encrypted email. Costing data sheets are requested corresponding to Action Periods associated with the Learning Collaborative (i.e., between Learning Sessions). The workbook documents financial and human capital resources directed toward the implementation of rapid ART start services and total monetary expenses and personnel hours dedicated to development and implementation of service models. Calculations include programmatic and structural expenses and personnel supported by RWHAP SPNS, as well as other in-kind sources. Examples of expenses included are: personnel effort to develop protocols; EHR/EMR modifications necessary for implementation; provider and staff trainings; routine performance monitoring and feedback; and personnel and care-related expenses above and beyond levels required before rapid ART start implementation.

##### Domains

The goal is to obtain a measure of the true *costs* to implement rapid ART start, inclusive of funding sources associated with this initiative and any other source. The costs of implementation do not include expenses and personnel dedicated to the development and implementation of local or multisite RWHAP SPNS evaluations, participation in Learning Collaborative and initiative-developed QI activities, or routine costs of care independent of rapid ART start.

### Analyses

In this mixed-methods study, we are using quantitative methods for understanding the implementation process and outcomes of the rapid start programs. Qualitative methods are critical to understanding team readiness and dynamics as they play out over time. Overall interpretation of our results will consist of an integration of qualitative and quantitative findings, using a modified Embedded Experimental Design [62].

Analyses of all qualitative data consist of organization and examination following the principles of thematic and Framework Analysis [63], useful for analysis of qualitative data when some *a priori* domains are defined based on the research questions of interest; for example elucidating the components of *implementation strategies* (Name It, Define It, Specify It [57]) and how they are influenced by domains outlined by DCF. Once *a priori* domains are defined, initial coding of the data consists of reviewing source documents and identifying sections of the text that correspond to the *a priori* domains and developing new domains as needed. To organize and sort data, all materials are entered into Dedoose (Version 5.0.11) a web-based qualitative data analysis platform. Excerpts associated with key codes are summarized and tabled for comparison and theme identification.

Analyses of our quantitative data will use SAS [64] statistical suite to employ logistic generalized estimating equations (GEE) to evaluate the quantitative relationship between components of the framework measured at the organizational and individual levels while controlling for repeated measures. Our primary analyses will identify implementation strategies, implementation outcomes including acceptability and feasibility, cost and cost effectiveness, and service outcomes that predict our primary client outcomes: viral suppression and durable viral suppression.

## Discussion

The clinical benefits of rapid ART start following diagnosis of HIV infection have been demonstrated in numerous studies, accelerating entry into care, shortening times to viral suppression, declining in morbidity, and preventing onward transmission. However, models for delivery of rapid ART start are not well studied or articulated in ways that provide practical guidance about how services should be organized and delivered to achieve maximum outcomes, or to reach diverse groups of patients, particularly those who are most marginalized in the health system, including sexual and racial minorities and those who are unhoused, have experience in carceral systems, use substances, or have a diagnosis of mental illness. Strategies remain untested, uncodified, and have not been disseminated to promote wide uptake of rapid ART start in all HIV care settings. Accelerating access to care and ART initiation, this initiative will help prevent the onward transmission of HIV, developing and testing models of care that will help newly diagnosed patients and those out of care quickly achieve viral suppression. This initiative’s work can reduce barriers to care for all patients, reducing disparities and increasing equity.

Current studies reflect specific contexts and populations and do not account for implementation strategies within service delivery models, nor for patient population contexts [65, 66]. These studies underscore the importance of embedding rapid ART start within carefully designed programs that not only offer medication but also attend to engagement in care and support the complex needs of people on lifelong ART to prevent attrition. Our evaluation will document the factors associated with implementation, with particular attention to organizational context [67]. Each of the implementation sites in this project is developing a unique protocol for delivery of rapid ART start that address details about service delivery, allocation of staffing, roles and responsibilities, clinic flow, and data collection systems among other implementation strategies. These protocols are iterative and reflect changes tested through QI cycles in their Learning Collaborative participation. The compilation of these successful tests of change, demonstrated through improved performance metrics of clinical outcomes, will contribute to a project capstone implementation guide that will reflect the best practices of implementation of rapid ART start across sites. Our systematic collection of data associated with clinic uptake and the documentation of changes required to implement rapid ART start for this evaluation has the potential to produce important information–in fact the blueprint–for the implementation of rapid ART start.

## Data Availability

Not applicable.

## References

[1] Centers for Disease Control and Prevention. HIV Diagnoses 2021 [updated June 2., 2021. Available from: https://www.hiv.gov/hiv-basics/overview/data-and-trends/statistics.

[2] Centers for Disease Control and Prevention. Monitoring selected national HIV prevention and care objectives by using HIV surveillance data—United States and 6 dependent areas., 2019. 2021. Contract No.: 2.

[3] Office of AIDS Research NIoH. Recommendations for the Use of Antiretroviral Agents in Adults and Adolescents with HIV. 2021 June 3, 2021.

[4] Pilcher C, Ospina-Norvell C, Dasgupta A, Jones D, Hartogensis W, Torres S (2017). The effect of same-day observed initiation of antiretroviral therapy on HIV viral load and treatment outcomes in a US public health setting. JAIDS J Acquir Immune Defic Syndr.

[5] Bacon O, Chin J, Cohen S, Sachdev D, Coffey S, Scheer S, et al. Decreased time from HIV diagnosis to care, ART initiation, and virologic suppression during the citywide RAPID initiative in San Francisco. Clinical Infectious Diseases; 2020. 10.1093/cid/ciaa620 PMC8561247 32449916

[6] Coffey S, Bacchetti P, Sachdev D, Bacon O, Jones D, Ospina-Norvell C (2019). RAPID antiretroviral therapy: high virologic suppression rates with immediate antiretroviral therapy initiation in a vulnerable urban clinic population. AIDS.

[7] Colasanti J, Sumitani J, Mehta C, Zhang Y, Nguyen M, del Rio C (2018). Implementation of a rapid entry program decreases time to viral suppression among vulnerable persons living with HIV in the Southern United States. Open Forum Infectious Diseases.

[8] Halperin J, Conner K, Butler I, Zeng P, Myers L, Clark R (2019). A Care Continuum of immediate ART for newly diagnosed patients and patients presenting later to care at a federally qualified health center in New Orleans. Open Forum Infectious Diseases.

[9] World Health Organization. Guideline on when to start antiretroviral therapy and on pre-exposure prophylaxis for HIV. Geneva, Switzerland; 2015. 26598776

[10] Saag M, Benson C, Gandhi R, Hoy J, Landovitz R, Mugavero M (2018). Antiretroviral drugs for treatment and prevention of HIV infection in adults: 2018 recommendations of the International Antiviral Society–USA panel. JAMA.

[11] Panel on Antiretroviral Guidelines for Adults and Adolescents. Guidelines for the Use of Antiretroviral Agents in Adults and Adolescents with HIV. 2019.

[12] Michienzi SM, Barrios M, Badowski ME (2021). Evidence regarding Rapid initiation of antiretroviral therapy in patients living with HIV. Curr Infect Dis Rep.

[13] Boyd M, Boffito M, Castagna A, Estrada V (2019). Rapid initiation of antiretroviral therapy at HIV diagnosis: definition, process, knowledge gaps. HIV Med.

[14] Ford N, Migone C, Calmy A, Kerschberger B, Kanters S, Nsanzimana S (2018). Benefits and risks of rapid initiation of antiretroviral therapy. AIDS.

[15] Martin TCS, Abrams M, Anderson C, Little SJ (2021). Rapid Antiretroviral Therapy among individuals with Acute and Early HIV. Clin Infect Dis.

[16] Bogdanić N, Bendig L, Lukas D, Zekan Å, Begovac J (2021). Timeliness of antiretroviral therapy initiation in the era before universal treatment. Sci Rep.

[17] Ilagan DJC, Eitniear L, Cole K, Duggan J, Sahloff E. Time between diagnosis and achievement of virologic suppression in people living with HIV. American journal of health-system pharmacy. 2021. 10.1093/ajhp/zxab269 34185842

[18] O’Shea JG, Gallini JW, Cui X, Moanna A, Marconi VC (2022). Rapid Antiretroviral Therapy Program: development and evaluation at a Veterans Affairs Medical Center in the Southern United States. AIDS Patient Care STDs.

[19] Al-Hayani AWM, Cabello-Úbeda A, Del Palacio-Tamarit M, Rodríguez-Alonso B, Carrillo-Acosta I, Álvarez-Álvarez B (2022). Initiation of antiretroviral therapy in treatment-naive adults with HIV infection at the first specialist appointment. J Antimicrob Chemother.

[20] Lifson A, Grund B, Gardner E, Kaplan R, Denning E, Engen N (2017). Improved quality of life with immediate versus deferred initiation of antiretroviral therapy in early asymptomatic HIV infection. AIDS.

[21] Cohen M, Chen Y, McCauley M, Gamble T, Hosseinipour M, Kumarasamy N (2016). Antiretroviral therapy for the Prevention of HIV-1 transmission. N Engl J Med.

[22] Estrada V, Górgolas M, Peña JA, Tortajada E, Castro A, Presa M (2022). Epidemiologic and economic analysis of Rapid Antiretroviral Therapy initiation with Bictegravir/Emtricitabine/Tenofovir Alafenamide in Spain. PharmacoEconomics - open.

[23] Borges A, Neuhaus J, Babiker A, Henry K, Jain M, Palfreeman A (2016). Immediate Antiretroviral Therapy reduces risk of infection-related cancer during early HIV infection. Clin Infect Dis.

[24] Herout S, Mandorfer M, Breitenecker F, Reiberger T, Grabmeier-Pfistershammer K, Rieger A (2016). Impact of early initiation of antiretroviral therapy in patients with acute HIV infection in Vienna, Austria. PLoS ONE.

[25] Thornhill J, Inshaw J, Kaleebu P, Cooper D, Ramjee G, Schechter M (2016). Brief report: enhanced normalization of CD4/CD8 ratio with earlier antiretroviral therapy at primary HIV infection. JAIDS J Acquir Immune Defic Syndr.

[26] Kamya MR, Petersen ML, Kabami J, Ayieko J, Kwariisima D, Sang N et al. SEARCH Human Immunodeficiency Virus (HIV) Streamlined Treatment Intervention Reduces Mortality at a Population Level in Men With Low CD4 Counts. Clinical infectious diseases. 2021. 10.1093/cid/ciaa1782 PMC8492199 33783495

[27] Mateo-Urdiales A, Johnson S, Nachega J, Eshun-Wilson I. Rapid initiation of antiretroviral therapy for people living with HIV. Cochrane Database of Systematic Reviews; 2018. 10.1002/14651858.CD012962.pub2 PMC6575156 31206168

[28] Pettit AC, Pichon LC, Ahonkhai AA, Robinson C, Randolph B, Gaur A et al. Comprehensive Process Mapping and Qualitative Interviews to Inform Implementation of Rapid Linkage to HIV Care Programs in a Mid-Sized Urban Setting in the Southern United States. Journal of acquired immune deficiency syndromes (1999). 2022;90(S1):S56-S64. 10.1097/QAI.0000000000002986 PMC9204789 35703756

[29] Harkness A, Wawrzyniak AJ, Kolber MA, Villamizar K, Botero V, Rodriguez JE et al. Multilevel Determinants of Rapid Antiretroviral Treatment Implementation and Demand in Miami-Dade County. Journal of acquired immune deficiency syndromes (1999). 2022;90(S1):S177-S89. 10.1097/QAI.0000000000002978 PMC9204784 35703770

[30] Beattie C, Wiewel E, Zhong Y, Brown P, Braunstein S, Farquhar X (2019). Multilevel factors Associated with a lack of viral suppression among persons living with HIV in a federally funded Housing Program. AIDS Behav.

[31] Dandachi D, May S, Davila J, Cully J, Amico K, Kallen M et al. 1770. The Association of Unmet Needs With Subsequent Retention in Care and HIV Suppression Among Hospitalized Patients With HIV Who Are Out of Care. Open forum infectious diseases. 2018;5(suppl_1):S65-S6. 10.1097/QAI.0000000000001874 PMC6289860 30272637

[32] Kimmel A, Masiano S, Bono R, Martin E, Belgrave F, Adimora A (2018). Structural barriers to comprehensive, coordinated HIV care: geographic accessibility in the US South. AIDS Care.

[33] Quinn K, Reed S, Dickson-Gomez J, Kelly J (2018). An exploration of syndemic factors that Influence Engagement in HIV Care among Black Men. Qual Health Res.

[34] Rebeiro P, Howe C, Rogers W, Bebawy S, Turner M, Kheshti A (2018). The relationship between adverse neighborhood socioeconomic context and HIV continuum of care outcomes in a diverse HIV clinic cohort in the Southern United States. AIDS Care.

[35] Jackson-Best F, Edwards N (2018). Stigma and intersectionality: a systematic review of systematic reviews across HIV/AIDS, mental illness, and physical disability. BMC Public Health.

[36] Logie C, Wang Y, Lacombe-Duncan A, Wagner A, Kaida A, Conway T (2018). HIV-related stigma, racial discrimination, and gender discrimination: pathways to physical and mental health-related quality of life among a national cohort of women living with HIV. Prev Med.

[37] Earnshaw V, Smith L, Chaudoir S, Amico K, Copenhaver M (2013). HIV Stigma Mechanisms and well-being among PLWH: a test of the HIV Stigma Framework. AIDS Behav.

[38] Jones J, Weiss K, Mermin J, Dietz P, Rosenberg E, Gift T, et al. Proportion of Incident HIV cases among men who have sex with men attributable to Gonorrhea and Chlamydia. A Modeling Analysis. Sexually transmitted diseases; 2019. 10.1097/OLQ.0000000000000980 PMC6530490 31095100

[39] Sanchez M, Scheer S, Shallow S, Pipkin S, Huang S. Epidemiology of the Viral Hepatitis-HIV Syndemic in San Francisco: A Collaborative Surveillance Approach. Public health reports (1974). 2014;129(1_suppl1):95–101. 10.1177/00333549141291S114 PMC3862995 24385655

[40] Cheng Q, Engelage E, Grogan T, Currier J, Hoffman R. Who Provides Primary Care? An Assessment of HIV Patient and Provider Practices and Preferences. J AIDS Clin Res. 2014;5(11). 10.4172/2155-6113.1000366 PMC4409003 25914854

[41] Petterson S, Liaw W, Phillips R, Rabin D, Meyers D, Bazemore A, Projecting. US Primary Care Physician Workforce Needs: 2010–2025. Annals of family medicine. 2012;10(6):503-9. 10.1370/afm.1431 PMC3495923 23149526

[42] Weiser J, Beer L, West B, Duke C, Gremel G, Skarbinski J (2016). Qualifications, demographics, satisfaction, and future capacity of the HIV Care Provider workforce in the United States, 2013–2014. Clin Infect Dis.

[43] Maiorana A, Steward W, Koester K, Pearson C, Shade S, Chakravarty D (2012). Trust, confidentiality, and the acceptability of sharing HIV-related patient data: lessons learned from a mixed methods study about Health Information exchanges. Implement science: IS.

[44] Zamudio-Haas S, Koester K, Maiorana A, Fuller S, Steward W, Gruber D (2019). Closing the Loop” developing state-level data sharing Interventions to promote optimum outcomes along the HIV Continuum of Care. AIDS Behav.

[45] Kalichman S, Kalichman M, Cherry C (2016). Medication beliefs and structural barriers to treatment adherence among people living with HIV infection. Psychol Health.

[46] Barbosu CM, Alcantara L, Sharma S, Marriott J, Babiy O, Deamer R et al. The Implementation of a State-wide Rapid Initiation of Antiretroviral Therapy as a Best Practice in Primary Care Practices Using an Academic Detailing Approach: Lessons Learned from New York State, United States. International journal of MCH and AIDS. 2022;11(1). 10.21106/ijma.539 PMC9083378 35601680

[47] Bacon O, Coffey S. Personal communication.

[48] Koester KA, Moran L, LeTourneau N, VanderZanden L, Coffey S, Crouch P-C (2022). Essential elements of and challenges to rapid ART implementation: a qualitative study of three programs in the United States. BMC Infect Dis.

[49] Rodriguez A, Wawrzyniak A, Tookes H, Vidal M, Soni M, Nwanyanwu R (2019). Implementation of an Immediate HIV treatment initiation program in a Public/Academic Medical Center in the U.S. South: the Miami Test and treat Rapid Response Program. AIDS Behav.

[50] The Breakthrough Series (2003). IHI’s collaborative model for achieving breakthrough improvement.

[51] Rohweder C, Wangen M, Black M, Dolinger H, Wolf M, O’Reilly C (2019). Understanding quality improvement collaboratives through an implementation science lens. Prev Med.

[52] DeLorenzo L, Fox J, Quinlivan E, Gilmore K, Ruetten M, Broaddus M (2019). Lessons learned from applying a modified learning collaborative model to promote change in Regional and Statewide HIV Care Systems. AIDS Behav.

[53] Schneider K, Agins B, Ng D, Monserrate J, Hirschhorn L (2012). Evaluation of regional HIV provider quality groups to improve care for people living with HIV served in the United States. J Health Care Poor Underserved.

[54] Helfat C, Finkelstein S, Mitchell W, Peteraf M, Singh H, Teece D (2007). Dynamic capabilities: understanding Strategic Change in Organizations.

[55] Leung RC (2012). Health information technology and dynamic capabilities. Health Care Manage Rev.

[56] Proctor E, Silmere H, Raghavan R, Hovmand P, Aarons G, Bunger A (2011). Outcomes for implementation research: conceptual distinctions, Measurement Challenges, and Research Agenda. Adm policy mental health mental health Serv Res.

[57] Proctor E, Powell B, McMillen J (2013). Implementation strategies: recommendations for specifying and reporting. Implement Sci.

[58] Steward W, Koester K, Collins S, Maiorana A, Myers J (2012). The essential role of reconfiguration capabilities in the implementation of HIV-related health information exchanges. Int J Med Inf (Shannon Ireland).

[59] Lusthaus C, Adrien M-H, Anderson G, Carden F, Montelván G. Organizational Assessment: a Framework for improving performance. Inter-American Development Bank, Washington DC; International Development Research Centre, Ottowa, Canada; 2002.

[60] Lombardi M, Spratling R, Pan W, Shapiro S (2018). Measuring Organizational Capacity to accelerate Health Care Innovation in Academic Health Centers. Qual Saf Health Care.

[61] Weiner B, Lewis C, Stanick C, Powell B, Dorsey C, Clary A (2017). Psychometric assessment of three newly developed implementation outcome measures. Implement Sci.

[62] Creswell JW, Plano Clark VL. Designing and Conducting Mixed Methods Research. Second edition Los Angeles and London: Sage Publications, 2011, pp xxvi, 4572011. p. xxvi.

[63] Goldsmith LJ (2021). Using Framework Analysis in Applied qualitative research. Qualitative Rep.

[64] SAS Institute Inc (2013). SAS® 9.4 statements: reference.

[65] Lilian RR, Rees K, McIntyre JA, Struthers HE, Peters RPH (2020). Same-day antiretroviral therapy initiation for HIV-infected adults in South Africa: analysis of routine data. PLoS ONE.

[66] Dah TTE, Yaya I, Mensah E, Coulibaly A, Kouamé JM, Traoré I (2021). Rapid antiretroviral therapy initiation and its effect on treatment response in MSM in West Africa. Aids.

[67] Horton T, Illingworth J, Warburton W. The spread challenge: How to support the successful uptake of innovations and improvements in health care. Long, UK; 2018.

